# Induced pluripotent stem cells as a tool to study brain circuits in autism-related disorders

**DOI:** 10.1186/s13287-018-0966-2

**Published:** 2018-08-23

**Authors:** Aline Vitrac, Isabelle Cloëz-Tayarani

**Affiliations:** 1 Human Genetics and Cognitive Functions, Institut Pasteur, 25 rue du Docteur Roux, 75015 Paris, France; 2 CNRS UMR 3571, Institut Pasteur, 25 rue du Docteur Roux, Paris, France; 3 Université Paris Diderot, Sorbonne Paris Cité, Human Genetics and Cognitive Functions, 25 rue du Docteur Roux, Paris, France

**Keywords:** Human induced pluripotent stem cells, Autism, Developmental disorders, Transplantation, Brain circuits

## Abstract

The mammalian brain is a very complex organ containing an estimated 200 billion cells in humans. Therefore, studying human brain development has become very challenging given all the data that are available from different approaches, notably genetic studies.

Recent pluripotent stem cell methods have given rise to the possibility of modeling neurodevelopmental diseases associated with genetic defects. Fibroblasts from patients have been reprogrammed into pluripotent stem cells to derive appropriate neuronal lineages. They specifically include different subtypes of cortical neurons that are at the core of human-specific cognitive abilities. The use of neurons derived from induced pluripotent stem cells (iPSC) has led to deciphering convergent and pleiotropic neuronal synaptic phenotypes found in neurodevelopmental disorders such as autism spectrum disorders (ASD) and their associated syndromes. In addition to these initial studies, remarkable progress has been made in the field of stem cells, with the major objective of reproducing the in vivo maturation steps of human neurons. Recently, several studies have demonstrated the ability of human progenitors to respond to guidance cues and signals in vivo that can direct neurons to their appropriate sites of differentiation where they become fully mature neurons.

We provide a brief overview on research using human iPSC in ASD and associated syndromes and on the current understanding of new theories using the re-implantation of neural precursors in mouse brain.

## Background

The use of induced pluripotent stem cells (iPSC) has provided new opportunities for analyzing brain development and the consequences of its dysfunctions in neurodevelopmental disorders. The iPSC approach has been particularly useful for neurodevelopmental diseases for which major genes are considered responsible. One important aspect resides in the fact that the reprogrammed iPSC studied so far carry single genetic deficits which were initially identified in the genomes of patients. This is the case for autism spectrum disorders (ASD) and their related disorders, which include Rett syndrome, Timothy syndrome, fragile X syndrome, and Phelan-McDermid syndrome (PMS). Causative genes are mostly related to synaptic functions. Among others, traditional approaches include large-scale genomic data analysis and engineered animal models [1–3]. The discovery of human iPSC has enabled the analysis of neuronal phenotypes after the derivation of patients’ somatic cells into neurons. One limitation of such an in vitro approach is the inability to grow the cells for periods long enough to reproduce the postnatal development and maturation of human iPSC-derived neurons. Alternative techniques are based on the use of neural progenitors that are patterned after their re-implantation in mouse brain to undergo differentiation via specific neural pathways in vivo.

## In vitro use of human iPSC-derived neurons in neurodevelopmental disorders: the case of syndromic and non-syndromic forms of ASD

A common understanding of the pathogenesis of neurodevelopmental disorders, including ASD, has still not been achieved. This remains an important issue, as it is a prerequisite for the development of pharmacological drugs for the treatment of the core symptoms of such disorders. Reprogramming of iPSC from patients into neuronal cell types was first used to further elucidate the phenotypes related to pathologies such as Alzheimer’s disease [4], Parkinson’s disease [5], epilepsy [6], and schizophrenia [7, 8]. Peripheral neurodegenerative disorders such as amyotrophic lateral sclerosis [9] have also been investigated. For all these pathological conditions, human iPSC have been reprogrammed into selective neuronal cell types by considering the neuronal phenotypes that are damaged as predicted by available animal models and clinical investigations in patients. The iPSC model has enabled further insights into the cellular and molecular mechanisms that are affected during brain development. This model was first developed in the case of monogenic diseases such as Rett syndrome, fragile X syndrome, Timothy syndrome, PMS, and various forms of schizophrenia that display symptoms in common with ASD. The common features between such monogenic diseases and ASD include cognitive dysfunctions with mental retardation and dysmorphia. Other features comprise epilepsy (atypical forms of Rett syndrome), cardiac dysfunction (Timothy syndrome), motor coordination, and sensorial hypersensitivity (fragile X syndrome). Studies using iPSC technology have been focused both on neural progenitors and on mature neurons derived mostly from the fibroblasts (or less often from blood) of patients. The identification of phenotypes in human iPSC-derived neurons was first studied in four syndromic forms of ASD with well-known identified causal genes.

Rett syndrome is a severe X-linked neurodevelopmental disorder. The main form of this disorder is characterized by defects in the *MECP2* gene coding for the transcriptional regulator methyl CpG binding protein 2, which is expressed in a wide variety of tissues, including the brain. The most extensive work using the iPSC model has been devoted to Rett syndrome, with the phenotypic characterization of these cell lines obtained from patients’ fibroblasts. Decreased cell soma size and neuritogenesis [10], reduced expression of the adhesion molecule L1 [10], and synaptic alterations [11, 12] have been observed by using this model, supporting the expected defects in neuronal connectivity.

Timothy syndrome is a very rare autosomal dominant disorder which results from mutations in the *CACNA1C* gene coding for the alpha-1 subunit of the L-type voltage-gated calcium channel Cav1.2. To date, only a few studies have analyzed the neuronal phenotypes of human iPSC-derived neurons, which have shown specific alterations in calcium signaling [13] and dendritic plasticity [14]. One of the initial studies reported a reduced number of cells expressing the DNA-binding protein SATB2 [15]. Satb2 regulates the fate of upper and deeper layers of cortical neurons as shown in mouse brain models [16]. It is therefore reasonable to suggest the existence of deleterious effects on neural development which are due to altered calcium signaling in parallel with the absence of SATB2 protein, and more specifically on cortico-cortical connections. Nevertheless, it should be noted that in vitro developmental neuronal patterns differ from in vivo ones, especially when the deleterious mutations target pleiotropic genes, such as *CACNA1C*, that coordinate organ functions in a non-independent manner.

PMS is a neurodevelopmental disorder strongly associated with ASD that is caused by a deletion of the *SHANK3* gene at the 22q13 locus, identified as a 22q13 deletion syndrome. Shcheglovitov and colleagues [17] used iPSC-derived cortical neurons from two patients and observed significant deficits in the excitatory transmission of reprogrammed neurons which were rescued by IGF1 exposure.

Fragile X syndrome is considered as one of the most common cause of syndromic ASD. This syndrome results from an expansion of a CGG repeat within the fragile X mental retardation 1 (FMR1) gene on the X chromosome. This gene is required for neuronal development and a deficiency in its corresponding protein leads to altered neuronal connectivity. It has been difficult so far to find a model to study this syndrome using iPSC technology. The more recent study by Doers and colleagues [18] clearly demonstrates reduced neurite outgrowth in neurons from patients. Reduced neurite outgrowth may alter axonal growth as well as the differentiation of presynaptic and postsynaptic components, and consequently may lead to alteration of short-range and long-range neuronal connectivity.

ASD are neurodevelopmental disorders characterized by deficits in social cognition, communication, and behavior as well as moderate to severe mental retardation. ASD have a complex genetic basis, with hundreds of identified candidate genes which cannot be individually responsible for ASD clinical features and cellular phenotypes. This complexity has led to considerable effort to identify functional pathways that may reveal cellular connections between the candidate genes. Among these, the glutamatergic pathway includes genes that, once mutated, are thought to be responsible for both syndromic and non-syndromic ASD [19]. Indeed, ASD present with alterations in the brain cortex and its development and morphological organization and the brain’s short-range and long-range connectivity [20]. For example, an abnormal connectivity between the cerebellum and the entire cerebral cortex has recently been shown using fMRI brain imaging [21]. The development and maintenance of neuronal networks depend on differentiation of neurons and axonal outgrowth as well as dendrite branching. We have shown that these processes are controlled differently by cell-adhesion molecules such as contactin 4 (CNTN4), contactin 5 (CNTN5), and contactin 6 (CNTN6) proteins [22]. Among the genes which code for CNTN4–6, we have demonstrated that *CNTN6* is a susceptibility gene for ASD [23]. Alterations in the formation of neural networks that are controlled by CNTN6 may underlie the cognitive, sensory, and motor deficits that we observe in autistic patients carrying *CNTN6* coding variants [22].

## Modeling shankopathies using human iPSC-derived neurons

Among the mutated genes in ASD, the *SHANK* genes offer one of the best possibilities to explore the core symptoms of these disorders by analyzing the cellular defects that are associated with mutations in them. The major *SHANK* family genes involved in ASD include *SHANK1*, *SHANK2*, and *SHANK3*. *SHANK* genes encode scaffolding proteins present at the postsynaptic density of excitatory synapses. The first report on specific *SHANK3* mutations was provided by our laboratory [24]. Further investigations have clearly demonstrated the role of all *SHANK* genes in ASD [1]. The involvement of *SHANK3* as a causal gene in ASD was observed in 0.7% of patients in large cohorts, with different types of mutations including microdeletions, point mutations, and stop mutations [1]. *SHANK3* is also associated with behavioral phenotypes of the 22q13 deletion syndrome [25, 26]. Human iPSC have been generated from patients with heterozygous deletions of chromosome 22q13.3. Derived neurons displayed reduced SHANK3 expression and major defects in their excitatory but not inhibitory synaptic transmission [17]. These findings strongly suggest that a disruption of the excitatory/inhibitory balance occurs in the brain of patients with PMS. Kathuria and colleagues [27] differentiated iPSC from two patients with ASD carrying microdeletions of *SHANK3* into either cortical or olfactory placodal neurons. These authors showed that placodal neurons had a reduced number of synapses compared with control neurons. The young postmitotic neurons also had reduced cell bodies with higher neuronal arborization. These two developmental phenotypes were specific to placodal neurons and were not observed in iPSC-derived cortical neurons. The morphogenetic deficits were rescued by genome editing techniques [27]. The iPSC model has also been used in patients with ASD presenting de novo point truncating mutations in the *SHANK3* gene; under the experimental conditions, pyramidal excitatory neurons accounted for more than 80% of cortical neurons [28]. In a subsequent study and under similar experimental conditions, these authors were able to evaluate and reverse the neuronal dysfunctions in two individuals with de novo point mutations in the *SHANK3* gene [29], namely decreased neurite length and branching and spontaneous calcium oscillations. In addition, the authors found that lithium as well as valproic acid and fluoxetine increased the *SHANK3* mRNA and protein levels in a concentration-dependent manner [29]. SHANK3 is expressed at neuronal excitatory synapses and forms protein complexes [30] which may contribute to ASD phenotypes when they are broken up. SHANK3 interactomes are expressed at single-spine level [31], which also receive neuronal excitation inputs. For the analysis of spine densities, most studies using animal models have been performed in two dimensions, which may not totally reflect the asymmetric morphology of diverse spine categories. Regarding ASD and human iPSC models, none of the published data have so far described a quantitative analysis of spinogenesis in patients with *SHANK3* mutations. Using the same protocol and human iPSC lines described by Darville and colleagues [29], we established a method which allows the quantification of spine morphology in three dimensions [32]. The shape of dendritic spines and volume vary according to the stage of their maturation. Mature spines are usually characterized by a larger head and a thin neck, whereas immature spines are thinner with a poorly defined head with small postsynaptic densities. We found the latter category to be predominant in human iPSC-derived pyramidal neurons from individuals without ASD-related disorders (unpublished observations). We are undertaking an extensive analysis of spinogenesis in a subset of patients carrying different de novo *SHANK3* point mutations to evaluate the inter-individual variability. This aspect is still lacking so far, since most studies have been conducted using cells reprogrammed from a few patients only.

## Main pitfalls using human iPSC cells in vitro

iPSC culture systems can offer an almost unlimited source of neurons for fundamental research on the first stages of neural development and for pharmacological screening. However, this model presents some limitations. Indeed, reprogramming of somatic cells through the expression of the four Yamanaka transcription factors, *OCT4*, *KLF4*, *SOX2*, and *cMYC*, has been shown to be asynchronous and have low efficiency. The rate of cell reprogramming also depends on donor cell types and culture conditions [33]. Different models have been proposed to analyze the reprogramming processes and the roles of transcription factors and epigenetic regulators [34]. To circumvent these problems, various methods have been developed in order to study reprogramming dynamics under more unified frameworks [35]. Alternative reprogramming protocols have also been proposed that are based on the use of synthetic capped mRNAs containing modified nucleobases (mod-mRNA) [36]. However, these methods do not seem to be efficient enough for the accurate reprogramming of human primary fibroblasts. A new, optimized method [37] which combines mod-mRNA with reprogramming factors together with improved cell culture conditions is encouraging and seems to provide an alternative approach for reprogramming of human fibroblasts in the case of ASD and related syndromes. New protocols have also been developed for improving the differentiation and maturation of iPSC-derived neurons [38].

When analyzing data from human iPSC-derived neurons in vitro, focusing on two-dimensional cell cultures often derived from one single cell type at a time may lead to an underestimation of cellular defects. For example, recent findings using the human iPSC model clearly indicate that astrocytes play important role in synaptogenesis and neuronal morphology [39]. The low efficiency of cell reprogramming observed so far has rendered the simultaneous derivation of distinct isogenic cell types from the same human iPSC much more difficult. The co-culture of several isogenic cell types, including distinct neuronal and microglial cells, would represent a significant improvement for studying ASD and its related disorders.

Finally, in vitro systems do not allow the reproduction of global cellular homeostasis and cell orientation and projections within the distinct cortical layers. It is also not clear to what extent the immature neurons that are produced in vitro recapitulate the diverse steps of neurogenesis. Interestingly, Imaizumi and colleagues [40] developed specific culture systems to control the identity of derived neuronal cells along the anteroposterior and dorsoventral axes. New protocols including three-dimensional culture systems [41] and brain organoids [42, 43] have been developed for iPSC models. Brain organoids consist of cellular aggregates derived from human embryonic stem cells (ESC) and iPSC. They may represent new in vitro systems with an oriented cell organization. Depending on the cell line and the number of passages, however, the brain organoids can be variable. Consequently, the development of brain organoids remains a challenging process due to the complexity of neuronal phenotypes and circuitry. So far, no method can provide a full reproduction of the phenotypic brain environment in vivo and the exact characteristics of developmental disorders due to the absence of a wide variety of conditions, including vascularization, nutrients, and specific developmental cues and signals. Human brain organoids are reviewed elsewhere in more detail [44, 45].

## Reconstruction of brain circuitry using neural transplants generated from iPSC

As discussed above, the main features of brain cortical development cannot be reproduced using in vitro models. Neurodevelopmental disorders are currently associated with cognitive dysfunctions, with the neocortex underlying high cognitive functions in humans. For this reason, cortical neuronal subtypes such as pyramidal glutamatergic cells have been predominantly used in vitro [15, 17, 28, 29, 46].

**Fig. 1:**
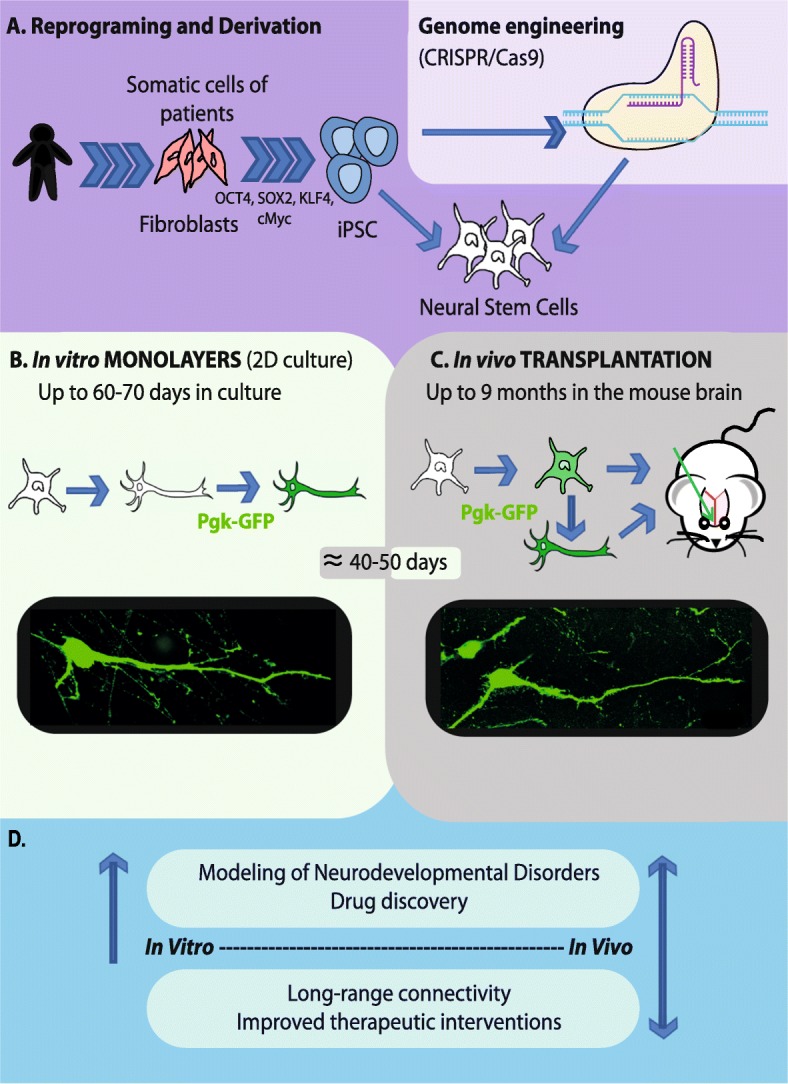
Main experimental designs for human iPSC models of monogenic neurodevelopmental disorders. **a** Patient’s specific iPSC are derived from fibroblasts using the four Yamanaka’s factors. Genome engineering using the CRISPR/Cas9 method allows the reversion of phenotypic defects by re-introducing the wild-type allele into the genome of iPSC lines. The CRISPR/Cas9 method also allows introduction of the mutation under study directly into the genome of control iPSC lines in order to compare visually similar phenotypes to those seen in the iPSC from patients. Both non-edited and isogenic iPSC are differentiated into the affected neuronal subtypes, mostly pyramidal cortical neurons in the case of cognitive disorders. **b** Viable neurons can be maintained in culture up to 70 days after the differentiation of neural stem cells (NSC). The transduction of neuronal cells with a green fluorescent protein (GFP)-lentivirus allows their visualization and phenotypic characterization using fluorescence microscopy. A GFP-labeled pyramidal neuron 40-45 days after the differentiation of NSC. **c** Neuronal precursors or neurons fully differentiated in vitro are transplanted into the brain of mouse neonates. The visualization of fluorescent neurons is done using fluorescent microscopy on brain slices. A transplanted GFP-labeled pyramidal neuron is illustrated at 40–50 days post-injection (picture from our experiments). Mice are maintained up to 9 months of age after grafting. **d** Comparative information and main therapeutics perspectives provided by the use of iPSC-derived neurons in vitro vs in vivo

For neurodevelopmental disorders, including ASD, defects in neuronal connectivity have been associated with increased local and reduced long-range connectivity as discussed above. One interesting aspect is the fact that an early neurodevelopmental dysfunction in single subcortical regions may modify the cerebral networks underlying early sensory-motor impairments and social deficits, including those observed in ASD [47]. The reconstruction of brain circuitry can be partially achieved by transplantation of human neurons generated from iPSC into mouse brain [48–52]. Since the early studies, significant progress has been made. Espuny-Camacho and colleagues [48] showed that ESC and iPSC can recapitulate corticogenesis and lead to sequential generation of functional pyramidal cells when grafted into mouse brain in vivo. In addition, these authors have demonstrated that, with regard to differentiation and connectivity, transplanted cells extend their ramifications over several months and constitute functional synapses with the host neuronal circuits [48]. Transplantation of neural precursors in mouse brain can be performed without a preliminary period of in vitro culture to allow cells to fully differentiate. Under such conditions, a post-characterization of cell phenotypes is necessary to clearly identify neurons from other iPSC-derived cells, such as oligodendrocytes and astrocytes. One key aspect resides in the fact that cortical pyramidal neurons derived from mouse or human iPSC follow species-specific maturation processes after their transplantation into mouse brain. Michelson and colleagues [49] also demonstrated that maturation of human ESC/iPSC takes 9 months post-transplantation and maintains the chronology of developmental steps for a given species. Using a similar approach, these authors derived neurons from mouse ESC in vitro which could be identified as those from visual cortex. The resulting neurons were then transplanted successfully into lesioned adult mouse visual cortex [49]. A possible rescue of the damaged pathways, including long-range and reciprocal axonal projections with appropriate synapses, was also observed [49]. Moreover, electrophysiological recordings were used to show that grafted neurons were responsive to visual stimuli [49]. Such an approach has not been tested with human iPSC. Nagashima and colleagues [50] developed a method consisting of an in utero transplantation system of pluripotent ESC based on a mild dissociation of adherens junctions in neuroepithelial tissue. Transplanted cells migrated from the subventricular zone to the cortical plate and, after only several days, presented the morphology of immature pyramidal cells [50]. To our knowledge, this method has not yet been used for the iPSC model. In their recent study, Falkner and colleagues [51] used chronic in vivo two-photon imaging to study the integration of mouse transplanted neurons into existing circuits of the mouse visual cortex [51]. After 2–3 months, the transplanted neurons were fully integrated, with functional properties indistinguishable from those of the pre-existing neuronal networks [51]. Functional imaging of grafted neurons into mouse brain represents an advantage compared to anatomical methods. This approach has not yet been used in the case of human neurons derived from patients with neurodevelopmental disorders. Figure 1 illustrates disease modeling using both in vitro and in vivo human iPSC models, independently or in a complementary manner. Figure 1 also represents the possible introduction of a genetic mutation by genome editing techniques such as CRISPR/Cas9 [53, 54] and/or the reversion of cellular phenotypic alterations found in monogenic disorders. The recent work by Wuttke and colleagues [52] is promising. Using an optogenetics-based electrophysiology approach, these authors demonstrated that developmentally “primed” cortical neurons can maintain their precise pattern of differentiation and regional connectivity after transplantation, with the development of appropriate long-distance projections and synapses. Reconstruction of the neonatal circuitry may be possible by the micro-transplantation of primed cortical neurons. Finally, methods for volume imaging of optically transparent brain tissues should offer new possibilities for the analysis of brain circuits.

The recent work by Mansour and colleagues is quite [54] promising. These authors have developed a method which consists of grafting human brain organoids into the adult mouse brain. They observed vascularized and functional intra-graft neuronal networks as well as graft-to-host synaptic connectivity. Together with ongoing technological improvements [45], grafting human brain organoids into mouse brain should represent an accurate alternative method to model a wide range of neurodevelopmental disorders, including ASD.

## Conclusions

The effective integration of transplanted cells that mature into neuronal subtypes together with appropriate long-range connectivity within critical regions such as brain cortex should allow the functional reconstruction of cortical circuitry over time. In addition, advances in genome editing technologies allow the genetic manipulation of iPSC in a site-specific manner. Combined with a circuit level analysis, such approaches should provide new plausible models to study human neurodevelopmental diseases and additional opportunities for future drug development.

## Abbreviations

ASD: Autism spectrum disorders
CNTN: Contactin
ESC: Embryonic stem cell
GFP: Green fluorescent protein
iPSC: Induced pluripotent stem cell
NSC: Neural stem cell
PMS: Phelan-McDermid syndrome
